# An evaluation of the therapeutic potential of fecal microbiota transplantation to treat infectious and metabolic diseases

**DOI:** 10.15252/emmm.201607035

**Published:** 2016-11-10

**Authors:** Albert K Groen, Max Nieuwdorp

**Affiliations:** ^1^ Department of Internal and Vascular MedicineAcademic Medical CenterAmsterdamThe Netherlands; ^2^ Department of PediatricsUniversity Medical CenterUniversity of GroningenGroningenThe Netherlands; ^3^ Department of Internal MedicineVUmc Diabetes CenterFree University Medical CenterAmsterdamThe Netherlands; ^4^ Wallenberg LaboratoryUniversity of GothenburgGothenburgSweden

**Keywords:** Metabolism, Microbiology, Virology & Host Pathogen Interaction, Pharmacology & Drug Discovery

## Abstract

Fecal microbiota transplantation (FMT) has had a long history in medicine for treating a number of human diseases. As early as during the 4^th^ century BC, FMT was used in China to treat patients with food poisoning and diarrhea. Over time, the method became obsolete, particularly after the realization that hygiene plays an important role in preventing infectious diseases. It was not until the late 1950s that FMT garnered interest again when the first reports about its use to treat fulminant enterocolitis appeared in the scientific literature. However, FMT's breakthrough as the method of choice for the treatment of persistent *Clostridium difficile* infection (CDI) came only after a double‐blind randomized trial (van Nood *et al*, 2013), which demonstrated 94% efficacy of FMT compared with 31% after conservative treatment with vancomycin.

FMT as a treatment modality has also been tested in a range of other diseases, such as inflammatory bowel disease (IBD), irritable bowel syndrome, obesity and metabolic syndrome, as well as neurological and psychological disorders, although most reports of the latter category are merely anecdotal. Yet, the results so far are not convincing. Reports about using FMT to treat IBD patients show considerable heterogeneity in terms of clinical effect (Moayyedi *et al*, 2015), whereas efficacy in patients with metabolic syndrome was transient. Insulin resistance improved 6 weeks after the FMT, but at 12 weeks, no significant effect was observed anymore on metabolic parameters and fecal microbiota composition (Vrieze *et al*, 2012).

The reason why FMT has such variable efficacy is probably due to the stability and resilience of the gut microbiota. CDI patients who have been treated multiple times with antibiotics have most of their bacterial diversity wiped out; FMT effectively repopulates this vacated space with a stable and healthy bacterial community (Girotra *et al*, 2016). In contrast, FMT recipients with metabolic syndrome were obese but otherwise healthy individuals with a stable and diverse fecal microbiota. Further investigation of why FMT failed to engraft novel species or change the gut microbiota in these naïve patients confirmed that their bacterial community is largely resilient to accepting new members in their midst. This “gated community” makes exceptions only for donor strains that are closely related—as identified via single nucleotide variants—to species that are already present in the patient's intestine (Li *et al*, 2016).

The regulatory factors that control a person's fecal microbial composition are not understood yet. A recent study addressed the important question of who sits at the gatehouse of the gut microbial community: the bacteria themselves, the host or both? Using a novel procedure called dissimilarity overlap curves (DOC), the authors looked at general relationships and cross‐talk between bacterial species independent of the host, which should predict the relative proportion of that species in the microbial community (Bashan *et al*, 2016). They indeed observed host‐independent cross‐talk, suggesting that the influence of the host, at least on the distribution of the major species, may be rather small. This conclusion also challenges any notion that the host immune system plays an important role in controlling the microbial community. Clearly, the study by Bashan and colleagues requires validation because of the potentially major consequences for engraftment with fecal microbiome‐based probiotics. If the influence of the host can really be ignored, it should be possible to develop generic procedures and products to manipulate microbiota composition, which would simplify probiotic therapy enormously.

Another explanation for the differences in the efficacy of FMT to treat various diseases is the tremendous variation in experimental protocols. Feces are prepared by different methods: dilution in saline, milk, water, or glycerol; different exposure time to the toxic oxygen environment; and varying modes of application all cause inconsistency. All these differences make it impossible to compare the results of different studies. Standard operation procedures for FMT are therefore urgently needed. Even when using standardized procedures, there is still the variable composition of donor and recipient microbiota that has to be dealt with. Donor health status will have to be screened extensively. Efficacy of frozen stool has been compared to fresh material in studies with CDI patients and no significant difference was observed (Lee *et al*, 2016), which provides the opportunity to characterize donor feces prior to application.

Moreover, in most studies published so far, the effects of FMT on human physiology were associated with bacterial composition. However, depending on the pre‐purification procedure, human feces contain considerably more than just bacteria. With the introduction of metagenomic analysis, it has become clear that, in addition to bacteria, the fecal microbiota contains a considerable amount of viruses, fungi, and phages as well as intact shedded colonocytes. A recent study reported that feces contained 10^11^ bacteria/g, 10^7^ intact colonocytes/g, 10^8^ viruses/g and 10^8^ archae/g. Although bacteria clearly dominate the gut population, it cannot be excluded that other components such as viruses and miRNAs influence host physiology (Liu *et al*, 2016).

To our knowledge, there have been no well‐documented serious adverse events after FMT reported since its rejuvenation in 1958. However, the lack of randomized, well‐controlled, and well‐documented studies does not exclude that such events have occurred. At the Academic Medical Center in Amsterdam, 600 FMTs have been carried out, mostly as research projects, and no adverse events have occurred so far. Given all the available data, we can conclude that FMT is a safe procedure. Nevertheless, a more optimal and safer way would either use standardized capsules from well‐screened donors feces (Youngster *et al*, 2014), or encapsulated well‐characterized mixtures of cultured microorganisms that have been proven to cure human disease. Unfortunately, this ideal situation is still far away from therapeutic application. As of now, no single bacterial strain or microorganism has been shown to be able to significantly impact human health. Interestingly, a combination of several novel probiotic bacterial strains has been shown to successfully treat patients with CDI, suggesting that this may be way to go (Khanna *et al*, 2016).

We speculate that improved delivery—for instance, by pH‐driven release from oral capsules—and more adequate oral dosing, which is currently limited to 10^12 ^CFU/day orally, of these novel probiotic strains might improve engraftment and efficacy. However, as previously mentioned, the residing gut microbial community is resilient to changes induced from the outside. Most probably, this could be overcome by sustained application of higher doses of a beneficial bacteria mixture or pretreatment with antibiotics. Future studies will have to ascertain whether these hypotheses hold true.

In addition, diet considerably influences fecal microbiota composition. Switching from a diet of animal‐derived products to one composed of plant‐derived products induces significant functional changes in microbiota composition. These changes are directly linked to, for instance, the type of fiber that has to be degraded, suggesting that substrate availability is a strong regulating factor in microbial composition and thus might be instrumental in engraftment of donor bacterial strains. Selecting the right diet for beneficial bacteria may pave the way for a more successful use of FMT.

In conclusion, further ongoing human research using FMTs will hopefully help to identify single or combination of bacterial strains that can be used as diagnostics or therapeutics. Moreover, these studies will show if fecal microbiota are indeed drivers of disease instead of being merely disease modifiers (Bonder *et al*, 2016). Other clinical factors that have not been addressed in these studies, such as ethnicity, are currently investigated in the AMC HELIUS study along with prospective follow‐up of participants (Stronks *et al*, 2013). Nevertheless, all of these identified bacterial strains should be repetitively identified in other cohorts comprising patients with the same disease. Also, there should be correlations with other biomarkers (e.g. like HbA1c) and these bacterial strains should prospectively be linked to disease outcome (such as development of *de novo* type 2 diabetes) in epidemiological studies (Fig 1). Only when these three criteria are fulfilled, it is expected that we can predict disease development in humans based on their microbiota and can be treated accordingly.

**Figure 1:**
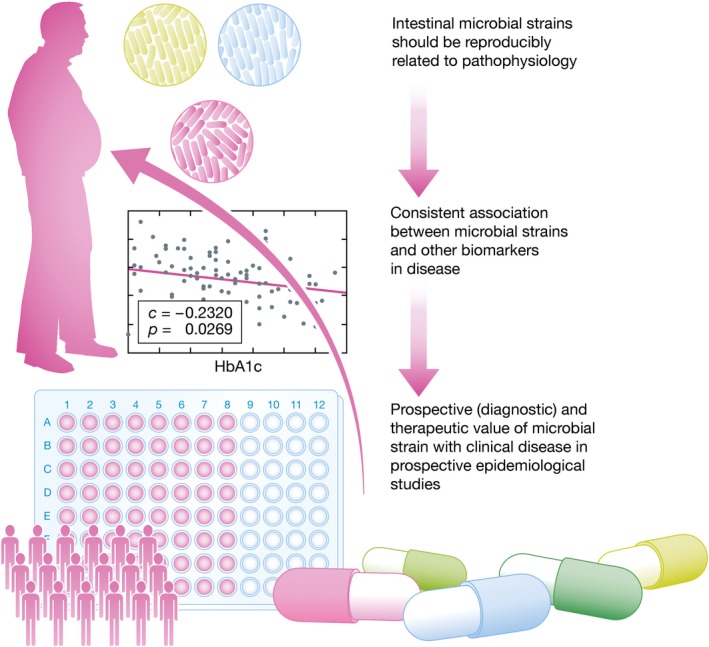
Schematic overview of identifying diagnostic and therapeutic bacterial strains from FMT

## Conflict of interest

M.N. is in the Scientific Advisory Board of Caelus Pharmaceuticals, the Netherlands, and in the Scientific Advisory Board of Seres Health, Boston, USA. None of these conflicts of interest is directly related to the research currently described.
